# The efficacy of a smartwatch-based haptic metronome in standardising chest compression rate: a crossover simulation study

**DOI:** 10.1016/j.resplu.2026.101389

**Published:** 2026-06-22

**Authors:** Mehmet Erdi Yılmaz, Talha Karahan

**Affiliations:** Department of Emergency Medicine, Kafkas University School of Medicine, Kars, Turkey

**Keywords:** Cardiopulmonary resuscitation, Chest compression rate, Haptic feedback, Smartwatch, Simulation, Metronome

## Abstract

**Background:**

High-quality chest compressions are crucial for effective resuscitation, yet maintaining the guideline-recommended rate of 100–120 compressions per minute is challenging, particularly in stressful and noisy prehospital environments. Haptic feedback delivered through wearable technology may offer a reliable alternative to auditory cues by providing tactile rhythm guidance.

**Objective:**

To evaluate the efficacy of a smartwatch-based haptic feedback system delivering 110 vibrations per minute in improving adherence to the target chest compression rate during simulated adult cardiac arrest, compared with unassisted cardiopulmonary resuscitation (CPR).

**Methods:**

In this prospective, experimental, simulation-based crossover study, 80 volunteers (paramedic students, intern physicians, emergency medicine residents, and laypersons; *n* = 20 each) performed one minute of self-paced chest compressions on a Laerdal Little Anne QCPR manikin and, after a five-minute rest, repeated the task with a Samsung Galaxy Watch 5 delivering haptic impulses at 110 bpm. The primary endpoint was the percentage of compressions performed within the 100–120 bpm target range; secondary endpoints were the proportion of participants achieving the target rate and self-reported user experience. The study was designed and is reported in accordance with the Reporting Guidelines for Health Care Simulation Research.

**Results:**

Haptic guidance substantially improved the percentage of compressions within the guideline-recommended 100–120 bpm range. The proportion of participants achieving the target compression rate increased from 45.0% at baseline to 93.75% with smartwatch-assisted feedback (McNemar test, *p* < 0.001). The mean percentage of compressions delivered within the target range rose from 43.55% during unassisted CPR to 90.71% with haptic guidance, and significant within-group improvements were observed across all four professional cohorts. Participant experience was highly positive: 93.75% endorsed the device for real-world clinical use, 95.00% found it useful for CPR training, and 95.00% expressed willingness to use it again.

**Conclusion:**

A smartwatch-based haptic metronome significantly improved adherence to the guideline-recommended chest compression rate across all experience levels. Given its high user acceptability and resilience to environmental noise, this wearable technology may provide a practical, low-cost adjunct to improve CPR quality in both training and prehospital settings.

## Highlights

• Smartwatch haptic feedback improves adherence to recommended CPR rates.
• Compression accuracy rose from 45% to 93.75% with 110 bpm haptic guidance.
• Performance gains were consistent across all professional experience levels.
• Smartwatch use was intuitive and caused minimal cognitive distraction.
• Wearable haptic systems offer a low-cost, noise-resilient feedback alternative.

## Introduction

Cardiac arrest remains a leading cause of mortality worldwide, and the quality of cardiopulmonary resuscitation (CPR) is a critical determinant of survival and favourable neurological outcomes.^1^ High-quality chest compressions are fundamental to effective resuscitation because they generate the coronary and cerebral perfusion pressures required to maintain myocardial and cerebral viability.^2^, ^3^, ^4^ Current international guidelines, including the 2025 European Resuscitation Council Guidelines and the 2025 International Liaison Committee on Resuscitation (ILCOR) Basic Life Support Consensus on Science with Treatment Recommendations, continue to endorse a compression rate of 100–120 per minute for adult CPR,^3^, ^4^ consistent with large observational datasets that report the highest survival within the 100–119 bpm range.^5^ Despite extensive educational efforts, rescuer fatigue, particularly during prolonged resuscitation, frequently leads to rapid deterioration in compression quality and makes guideline-adherent performance difficult to sustain.^6^

To address these challenges, several real-time feedback modalities have been developed, including visual cues, auditory metronomes and integrated audiovisual devices.^7^ A recent scoping review on CPR feedback technologies concluded that real-time devices consistently improve compression rate and depth in both training and clinical contexts, although effectiveness varies according to the sensory channel used and the environment in which the device is deployed.^8^ Although feedback devices generally improve CPR performance in controlled simulation settings, the chaotic and high-decibel nature of actual prehospital resuscitation can diminish the efficacy of auditory guidance, and certain visual interfaces have been reported to produce cognitive overload or distraction.^9^, ^10^

Haptic (vibration-based) feedback has emerged as a promising alternative in noisy or visually demanding environments because tactile signals are conveyed through a distinct sensory channel that is less susceptible to ambient interference.^11^ Experimental data indicate that tactile response times remain stable under increasing noise levels, whereas auditory performance degrades.^12^ Recent simulation studies of wearable technologies, particularly smartwatches, suggest that haptic cues can improve compression rate consistency and yield more uniform performance.^11^, ^13^

The aim of this study was to evaluate the efficacy of a smartwatch-based haptic feedback system, set at 110 vibrations per minute, in increasing the proportion of chest compressions performed within the recommended rate range. Performance with haptic guidance was compared with unassisted CPR at the rescuer’s self-selected rhythm in a simulated adult cardiac arrest scenario involving paramedic students, intern physicians, emergency medicine residents and laypersons.

## Materıals and methods

### Study design and setting

This prospective, experimental, simulation-based study was conducted at the Department of Emergency Medicine Simulation Laboratory of Kafkas University. The primary aim was to evaluate the efficacy of a haptic (vibration-based) smartwatch metronome in standardising chest compression rate during CPR. A within-subjects crossover design was used to minimise inter-individual variability. The protocol was conducted in accordance with the Declaration of Helsinki and was approved by the Kafkas University Faculty of Medicine Non-Interventional Clinical Research Ethics Committee (Decision No. KAÜ-TFEK 2025/07/07; 24 September 2025). The study was designed and is reported in accordance with the Reporting Guidelines for Health Care Simulation Research, and the completed checklist is provided as a supplementary file.^14^

### Participants

A total of 80 volunteers were enrolled and allocated to four cohorts of 20 participants representing different levels of medical expertise: paramedic students, medical students serving as intern physicians, emergency medicine residents, and laypersons with no formal medical training. Eligible participants were aged 18 years or older, agreed to participate voluntarily, and had no musculoskeletal or neurological limitation that would interfere with CPR performance. Individuals who had previously participated in metronome-guided CPR research, or who were physically unable to perform one minute of uninterrupted chest compressions, were excluded. Written informed consent was obtained from every participant.

### Smartwatch-based haptic metronome

**Fig. 1:**
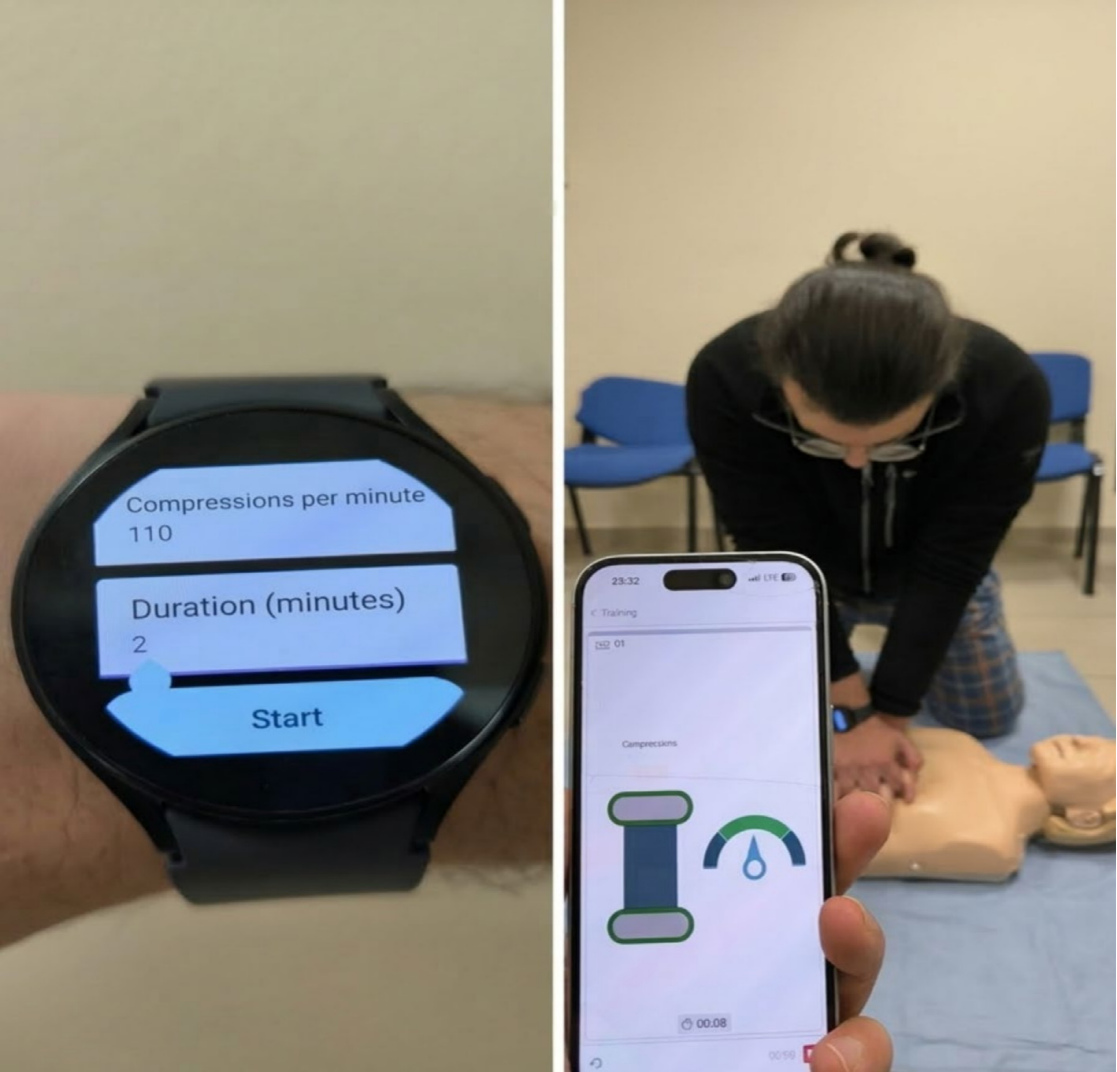
**Application of the smartwatch-based haptic feedback system during chest compressions. The Samsung Galaxy Watch 5, programmed to deliver vibrations at 110 bpm, is secured with a tightened strap to the wrist of the rescuer’s top (superior) hand while chest compressions are performed on the Laerdal Little Anne QCPR manikin**.

A proprietary application was developed for the Samsung Galaxy Watch 5 (Samsung Electronics, Suwon, South Korea) and programmed to deliver continuous haptic impulses at a fixed rate of 110 beats per minute, corresponding to the midpoint of the 100–120 bpm range recommended by current international guidelines.^3^, ^4^, ^15^ The vibration intensity was set to maximum, and the watch was secured to the wrist of the rescuer’s top (dominant or superior) hand with a tightened strap to prevent displacement during compressions (Fig. 1).

### CPR procedure

Compression performance was recorded with the Laerdal Little Anne QCPR manikin (Laerdal Medical, Stavanger, Norway), which provides objective digital feedback on rate, depth, recoil and overall compression quality. Before testing, all participants received a standardised verbal briefing on high-quality CPR (rate, depth, recoil and minimisation of interruptions), a practical demonstration, and a short orientation to the smartwatch and its haptic output. Each participant first performed one minute of chest compressions at their self-selected rhythm without any external feedback, then rested for a standardised five-minute washout interval to mitigate physical fatigue, and finally completed a second one-minute trial guided by the 110 bpm haptic metronome. The unassisted trial always preceded the guided trial to avoid carry-over of the entrainment effect from the metronome to the baseline measurement.

### Outcomes and data collection

All metrics were captured automatically by the Laerdal QCPR system. The pre-specified primary outcome was the percentage of chest compressions performed within the guideline-recommended target range of 100–120 bpm. Secondary outcomes were the proportion of participants who achieved the target rate, inter-group differences in performance, and participant-reported user experience. After both trials, participants completed a six-item structured questionnaire scored on a five-point Likert scale (1 = strongly disagree, 5 = strongly agree). The questionnaire assessed ease of use and physical comfort, perceived contribution to rhythmic accuracy, potential for cognitive distraction, and willingness to use the device in clinical prehospital practice and in CPR training.

### Statistical analysis

Data were analysed with SPSS for Windows, version 22.0 (IBM Corp., Armonk, NY). Normality was assessed using the Shapiro–Wilk test together with inspection of skewness and kurtosis. Inter-group comparisons were performed by one-way analysis of variance (ANOVA) with Bonferroni post-hoc correction, and within-subject differences between the unassisted and haptic-guided conditions were evaluated by repeated-measures ANOVA. Categorical variables were compared using the chi-square and McNemar tests. A two-sided p-value of less than 0.05 was considered statistically significant.

## Results

### Participant characteristics

Demographic characteristics of the study population are summarised in Table 1. The cohort (*N* = 80) comprised 39 men (48.75%) and 41 women (51.25%), with equal numbers (*n* = 20) of paramedic students, intern physicians, emergency medicine residents and laypersons. The mean age was 27.50 ± 7.22 years.

**Table 1:** Descriptive characteristics of participants (*N* = 80).

**Variable**	**Category**	***n***	**%**
Sex	Male	39	48.75
	Female	41	51.25

Profession	Paramedic Students	20	25.00
	Intern physicians	20	25.00
	Emergency medicine residents	20	25.00
	Laypersons	20	25.00

Age, mean ± SD (years)	All participants	27.50 ± 7.22	

Values are *n* (%) unless otherwise stated. SD: standard deviation.

### Primary outcome: compression rate accuracy (AP1 vs AP2)

**Fig. 2:**
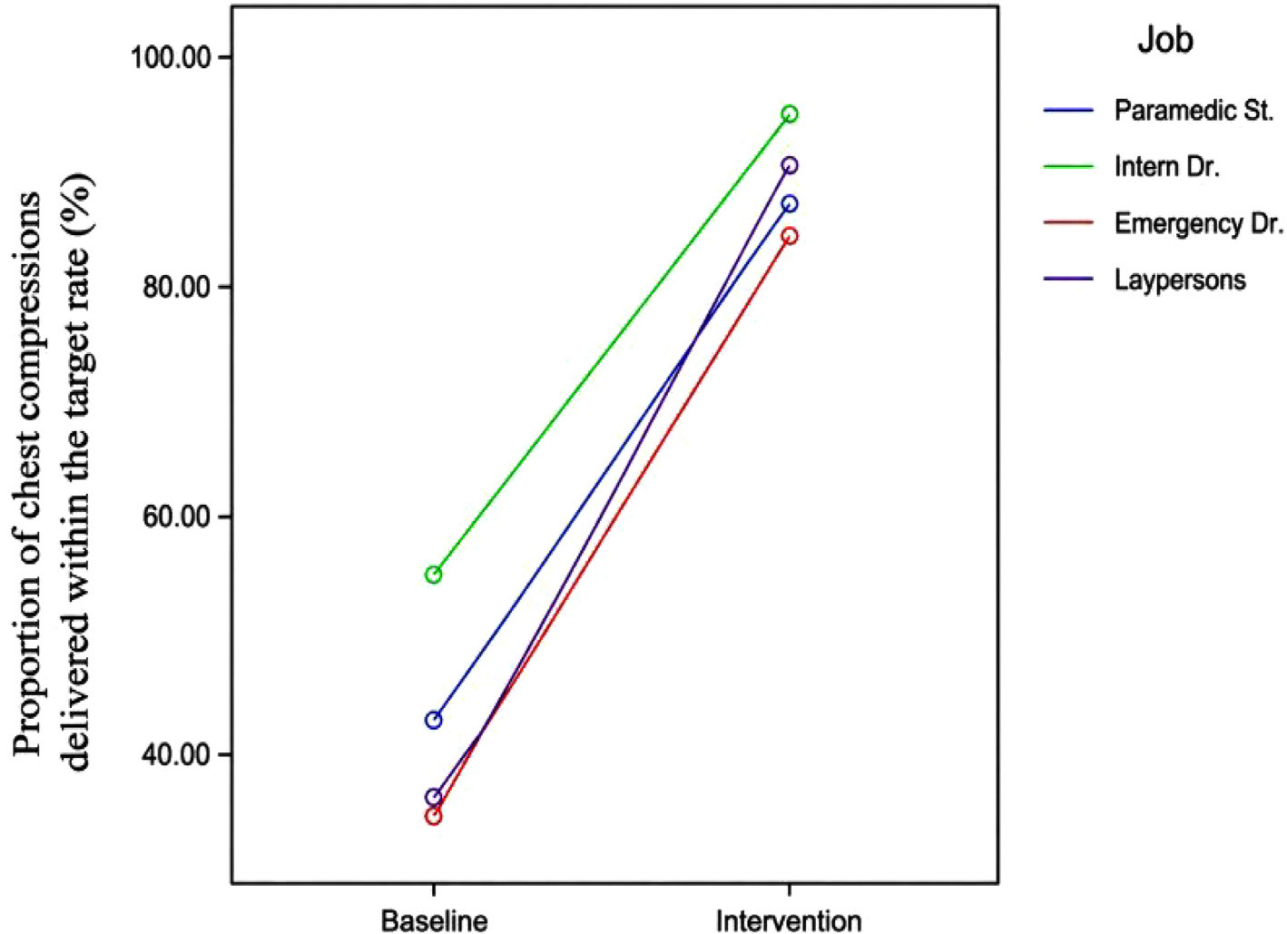
**Proportion of chest compressions delivered within the target rate of 100–120 compressions per minute, by professional group, during baseline unassisted CPR (Baseline) and during smartwatch-based haptic-guided CPR at 110 bpm (Intervention). Data are presented as group means for paramedic students, intern physicians, emergency medicine residents, and laypersons (*n* = 20 per group)**.

Repeated-measures ANOVA demonstrated a significant main effect of condition (baseline vs haptic-guided), confirming that haptic feedback substantially improved the percentage of compressions performed within the 100–120 bpm range (*F* = 95.86, *p* < 0.001, *η*^2^*_p_* = 0.56). Mean accuracy rose from 43.55 ± 41.87% at baseline (AP1) to 90.71 ± 20.21% with smartwatch guidance (AP2). The performance gain was consistent across all professional cohorts, as illustrated in Fig. 2 and detailed in Table 2.

**Table 2:** Percentage of compressions within the target rate (100–120 bpm), by professional group and condition (mean ± SD).

**Condition**	**Group**	**Mean accuracy ± SD (%)**	***n***	**Within-group Δ (AP2 − AP1), pp**
Baseline, unassisted (AP1)	Paramedic students	43.65 ± 40.21	20	+45.50*
	Intern physicians	56.10 ± 44.63	20	+38.70*
	Emergency medicine residents	37.60 ± 43.85	20	+49.50*
	Laypersons	36.85 ± 38.82	20	+54.95*
	Total	43.55 ± 41.87	80	+47.16*

Haptic-guided (AP2)	Paramedic students	89.15 ± 15.20	20	–
	Intern physicians	94.80 ± 13.02	20	–
	Emergency medicine residents	87.10 ± 27.24	20	–
	Laypersons	91.80 ± 22.80	20	–
	Total	90.71 ± 20.21	80	–

**Repeated-measures ANOVA**				

**Effect**	***F***	***p*-value**	**Partial *η*^2^**	

Time (AP1 vs AP2)	95.86	<0.001	0.56	
Group	1.07	0.37	–	
Time × Group	0.50	0.68	–	

Values for accuracy are means ± standard deviation (SD). AP1: accuracy percentage during baseline unassisted CPR. AP2: accuracy percentage during smartwatch-guided CPR at 110 bpm. pp: percentage points.

* *p* < 0.05 for paired within-group comparison (AP2 vs AP1).

The between-group effect was non-significant (*F* = 1.07, *p* = 0.37), indicating comparable performance among the four cohorts under both conditions, and the time × group interaction was also non-significant (*F* = 0.50, *p* = 0.68), suggesting that the magnitude of improvement attributable to haptic guidance was uniform across experience levels. Pairwise comparisons showed significant within-group improvements of 45.50 percentage points among paramedic students, 38.70 among intern physicians, 49.50 among emergency medicine residents and 54.95 among laypersons (all *p* < 0.05).

### Primary outcome: target rate achievement

The proportion of participants achieving the guideline-recommended rate of 100–120 bpm by professional group is shown in Table 3. At baseline, no significant difference was observed between groups (*χ*^2^ = 2.82, *p* = 0.42), with target-rate achievement ranging from 35.0% to 60.0%. With haptic guidance, achievement was uniformly high (*χ*^2^ = 0.64, *p* = 0.58): 95.0% of paramedic students, 95.0% of intern physicians, 90.0% of emergency medicine residents and 95.0% of laypersons. For the cohort as a whole, the proportion of participants reaching the target rate rose from 45.0% (*n* = 36) at baseline to 93.75% (*n* = 75) with smartwatch support (McNemar test, *p* < 0.001).

**Table 3:** Proportion of participants achieving the guideline-recommended chest compression rate (100–120 bpm), by professional group and condition.

**Condition**	**Paramedic students, *n*/*N* (%)**	**Intern physicians, *n*/*N* (%)**	**Emergency medicine residents, *n*/*N* (%)**	**Laypersons, *n*/*N* (%)**	**Overall, *n*/*N* (%)**	**Between-group *χ*^2^; *p***
Baseline, unassisted (CCPM1)	9/20 (45.00)	12/20 (60.00)	7/20 (35.00)	8/20 (40.00)	36/80 (45.00)	2.82; 0.42
Haptic-guided at 110 bpm (CCPM2)	19/20 (95.00)	19/20 (95.00)	18/20 (90.00)	19/20 (95.00)	75/80 (93.75)	0.64; 0.58

**Within-subject comparison (CCPM1 vs CCPM2):** McNemar test, *p* < 0.001.

Values are *n*/*N* (%). CCPM1: target-rate achievement at baseline (unassisted). CCPM2: target-rate achievement with smartwatch-based haptic guidance at 110 bpm. *χ*^2^ statistics compare proportions across the four professional groups within each condition; the McNemar test compares paired proportions between conditions for the cohort as a whole.

### Secondary outcome: participant experience

Participant responses to the post-intervention questionnaire are illustrated in Supplementary Fig. S1, while detailed subgroup questionnaire responses are presented in Supplementary Table S1. Overall, participant ratings were highly favourable. A total of 86.25% of participants agreed that the watch vibration made it easier to control compression rhythm, and 82.50% reported that the vibrations were easy to feel and follow. The device was rarely perceived as distracting, with only 7.50% of participants endorsing this reverse-coded item. A clear majority endorsed the device for real-world clinical use (93.75%) and for CPR training (95.00%), and 95.00% expressed willingness to use the device again.

## Dıscussion

This study demonstrates that a smartwatch-based haptic feedback system delivering 110 vibrations per minute significantly improves adherence to the recommended chest compression rate across a heterogeneous cohort of rescuers. The proportion of participants achieving the target rate increased from 45.0% at baseline to 93.75% with haptic guidance, an absolute improvement of 48.75 percentage points, and the effect was consistent in paramedic students, medical interns, emergency medicine residents and laypersons. These data suggest that haptic metronomes delivered through wearable technology can be effective regardless of prior clinical experience.

Our findings are consistent with a growing body of evidence supporting wearable haptic feedback for optimising CPR quality. LaPrad et al. reported that a smartwatch-based device increased compression efficiency to 87.5% among both healthcare providers and untrained rescuers, compared with 30.1% without feedback.^16^ Choi et al. likewise observed that haptic feedback significantly improved compression rate adherence (from 25.4% to 63.5%), particularly among initially low-performing participants.^13^ The higher accuracy achieved in our study (93.75%) may reflect the use of a fixed 110 bpm target, which sits at the midpoint of the recommended 100–120 bpm range and provides a clear, achievable rhythmic anchor.^3^, ^4^

Clinical data are broadly congruent with these simulation findings. Lyngby et al., in a study of 1545 patients with out-of-hospital cardiac arrest, observed that guideline-compliant compression rates rose from 60.2% to 74.6% when real-time feedback was provided.^17^ Wattenbarger et al. similarly reported that combining targeted training with real-time feedback increased the proportion of compressions within the target range from 41% to 81% among healthcare professionals.^18^ Our results extend this literature by showing that a commercially available smartwatch, delivering isolated haptic feedback at a fixed 110 bpm, can achieve comparable efficacy.

The efficacy of haptic feedback is biologically plausible. Unlike auditory or visual cues, vibration provides a continuous, non-intrusive temporal signal that does not compete for the rescuer’s visual or auditory attention, an advantage in the high-decibel and visually complex environment of prehospital resuscitation.^10^, ^19^ Whereas auditory metronomes can be masked by ambient noise, vibration-based feedback remains perceptible.^20^ Haptic cues also distribute cognitive load across sensory channels^21^, ^22^; consistent with the optimal theory of motor learning, an external focus delivered via tactile guidance may facilitate more automatic movement control^23^ and free attentional resources for chest recoil, airway management and team coordination.

A stable 110 bpm rhythm may also counteract the physiological consequences of fatigue, in which excessive rates compromise depth and recoil while insufficient rates reduce coronary and cerebral perfusion. A fixed tactile pacemaker can dampen the rate drift typically seen during prolonged resuscitation.^24^, ^25^ Although some authors have suggested a ceiling effect in which experienced providers benefit less from feedback,^13^ we observed significant improvements across all cohorts, indicating that even experienced emergency medicine residents can reach higher consistency when supported by wearable haptic technology.^26^

From a practical perspective, the high level of participant acceptability is notable. Only 7.50% of participants found the device distracting, whereas 93.75% endorsed it for real-world clinical use (Supplementary Fig. S1), suggesting that the technology could be integrated into both training curricula and clinical practice; participants’ enthusiasm for repeated use (95.00%) reinforces its potential as a low-cost educational adjunct. CPR quality is known to deteriorate during real-world resuscitation, with documented declines in compression depth over time and rates frequently falling below recommended thresholds during in-hospital arrests.^27^, ^28^, ^29^ Wearable feedback systems that supply a continuous external rhythm therefore offer a plausible mechanism to sustain compression quality, although field-based studies are required to confirm clinical benefit in the more demanding prehospital environment.

## Lımıtations

Several limitations should be acknowledged. First, the simulation-based design using a manikin cannot fully reproduce the physiological, environmental and psychological stressors of real cardiac arrest. Second, because the protocol used a fixed target of 110 bpm, corresponding to the midpoint of the current guideline range, we did not evaluate the relative efficacy of other rates within the 100–120 bpm window. Third, the study focused exclusively on compression rate; other determinants of CPR quality such as depth, chest recoil and hand position were not assessed. Fourth, the one-minute trial duration limits inferences about performance during prolonged resuscitation in which fatigue and skill decay become important. Finally, sequence randomisation was not used because the unassisted trial intentionally preceded the guided trial to avoid metronome carry-over to baseline performance; the clinical utility of the system in the field therefore remains to be confirmed under conditions that include ambient noise, patient transport and multitasking demands.

## Conclusion

In this simulation-based crossover study, a smartwatch-based haptic metronome improved adherence to the guideline-recommended chest compression rate, and this improvement was observed across paramedic students, intern physicians, emergency medicine residents, and laypersons. Combined with high user acceptability, low perceived distraction, and the accessibility and low cost of commercial smartwatches relative to dedicated resuscitation adjuncts, these findings indicate that wearable haptic feedback is a pragmatic option for standardising compression rate in both training and prehospital practice. The results support the incorporation of smartwatch-based haptic feedback into CPR education and further field-based evaluation, including bystander resuscitation, where conventional auditory and visual feedback may be limited by environmental conditions.

## CRediT authorship contribution statement

**Mehmet Erdi Yılmaz:** Writing – review & editing, Writing – original draft, Visualization, Validation, Supervision, Software, Resources, Project administration, Methodology, Investigation, Funding acquisition, Formal analysis, Data curation, Conceptualization. **Talha Karahan:** Writing – review & editing, Writing – original draft, Visualization, Validation, Supervision, Software, Resources, Project administration, Methodology, Investigation, Funding acquisition, Formal analysis, Data curation, Conceptualization.

## Funding

This research did not receive any specific grant from funding agencies in the public, commercial, or not-for-profit sectors.

## Ethical approval

The protocol was conducted in accordance with the Declaration of Helsinki and received formal approval from the Kafkas University Faculty of Medicine Non-Interventional Clinical Research Ethics Committee (Decision No: KAÜ-TFEK 2025/07/07; Date: September 24, 2025).

## Data availability statement

The data that support the findings of this study are available from the corresponding author upon reasonable request.

## Employment and affiliations

The authors are affiliated with the Department of Emergency Medicine, Kafkas University School of Medicine, and this affiliation has not influenced the objectivity of the study.

## Financial relationships

No author has received fees for consulting, lectures, or other forms of remuneration from entities related to the technology (Samsung) or resuscitation equipment (Laerdal) used in this study.

## Other interests

There are no patents, copyrights, or software royalties related to the smartwatch application described in this manuscript that would constitute a conflict of interest.

## Declaration of competing interest

The authors declare that they have no known competing financial interests or personal relationships that could have appeared to influence the work reported in this paper.

## Appendix A Supplementary material

Supplementary Data 1 **FiXXXg. S1:** Distribution of participant responses to the post-intervention smartwatch usability questionnaire (*N* = 80). Stacked bar charts display the percentage of responses in each Likert category (1 = Strongly Disagree to 5 = Strongly Agree) for each of the six questionnaire items. The reverse-coded item (“The device distracted me”) is marked with a dagger (†); for this item, low scores indicate absence of distraction.

Supplementary Data 2 The STROBE reporting checklist.
