# Supplementary material for: The efficacy of a smartwatch-based haptic metronome in standardising chest compression rate: a crossover simulation study
Source: Resusc Plus. 2026 Jun 22;30:101389. doi: 10.1016/j.resplu.2026.101389 (PMC13351885; doi:10.1016/j.resplu.2026.101389)
Supplement: Supplementary Data 1 — FiXXXg. S1: Distribution of participant responses to the post-intervention smartwatch usability questionnaire (N = 80). Stacked bar charts display the percentage of responses in each Likert category (1 = Strongly Disagree to 5 = Strongly Agree) for each of the six questionnaire items. The reverse-coded item (“The device distracted me”) is marked with a dagger (†); for this item, low scores indicate absence of distraction. [file mmc1.docx]

**Supplementary Figure S1: Likert scale participant survey responses.**

**Supplementary Table S1. Comparison of Participant Assessment Statements Following CPR Performance Across Professional Groups**

|  | Paramedic Students ^1^ Med(IQR) | Intern Physicians^2^ Med(IQR) | Emergency Physicians^3^ Med(IQR) | Laypersons^4^ Med(IQR) | KW;p | Difference (Bonferroni) |
| --- | --- | --- | --- | --- | --- | --- |
| The watch's vibration made it easier for me to control my CPR rhythm | 5.0(5.0-5.0) | 5.0(4.0-5.0) | 4.5(3.0-5.0) | 5.0(5.0-5.0) | KW:15,28;p:<0.01 | 3<1.4 |
| I could feel and follow the watch's vibration | 5.0(5.0-5.0) | 4.0(4.0-5.0) | 3.5(3.0-4.5) | 5.0(4.0-5.0) | KW:19,31;p:<0.01 | 3<1.4 |
| The device distracted me | 1.0(1.0-1.0) | 1.0(1.0-1.5) | 2.0(1.0-2.5) | 1.0(1.0-1.0) | KW:14,84; p<0.01 | 3>1.4 |
| This method could also be useful in real-life scenarios | 5.0(5.0-5.0) | 5.0(4.5-5.0) | 5.0(4.0-5.0) | 5.0(5.0-5.0) | KW:11,80; p<0.01 | 3<4 |
| I find this method useful in CPR training | 5.0(5.0-5.0) | 5.0(5.0-5.0) | 5.0(4.0-5.0) | 5.0(5.0-5.0) | KW:10,32; p<0.01 | 3<4 |
| I would like to use this type of method again | 5.0(5.0-5.0) | 5.0(5.0-5.0) | 5.0(4.0-5.0) | 5.0(5.0-5.0) | KW:11,98; p<0.01 | 3<1.4 |

KW-Kruskal Wallis-H test
